# Supplementary material for: Patterns of Intron Gain and Loss in Fungi
Source: PLoS Biol. 2004 Nov 30;2(12):e422. doi: 10.1371/journal.pbio.0020422 (PMC532390; doi:10.1371/journal.pbio.0020422)
Supplement: Table S1 — Also available at http://genes.mit.edu/NielsenEtAl/. (4.3 MB ZIP). [file pbio.0020422.st001.zip › NielsenEtAl/html/1246.html]

AN1211.1.NCU02746.1.MG04827.1.FG01152.1


```
 CLUSTAL W (1.82) Multiple Sequence Alignments - Introns Inserted


Sequence 1: NCU02746.1	481 aa
Sequence 2: FG01152.1	479 aa
Sequence 3: MG04827.1	483 aa
Sequence 4: AN1211.1	479 aa
Alignment Length: 498 aa
Number Identitical Residues: 227 aa
Alignment Score (without introns) 11459


MG04827.1 	---MALDPPTYLASLQNNIRQRPIPWDGAVRTGVLTEEQLAKTRAVEKPNR-DARKQTVE
NCU02746.1	---MSLDPPTYLASLQSNIRQRPIPWDGAVRAGTLTEEQLAKIRAVDKVKR-DVRRQVIE
FG01152.1 	---MSLDPPTYLASLQSNIRQRPIPWDGAVRAGTLTEEQYAKIRAVDKAKKPEQRKEIVS
AN1211.1  	MSAASLEPPTYLSSLQNNIRARPIPWEGAVRAGNITDDHLKKIKAVDKVRK-EQRRQTID
          	 :: :*:*****:***.*** *****:****:* :*:::  * :**:* .: : *:: :.

MG04827.1 	ADLDGYRALFVGEPGRPSVLESSNKP--IVQYLLVLLSDLLDC1VPTLSKALFKDS--DP
NCU02746.1	ANLDGYKTLFAGNNGQRSVLELASKRPDVCQYLLVLLSDLLDD1VPALSKAFFQSEDSNP
FG01152.1 	GDIDGYRVLFVGDEGKTSVLETAGKHANVIQYILVLLGDLLEA1VPSLAKALFKTK--EP
AN1211.1  	GDIPGYVTLLAGGSSGKSVLESAARRSDIVQYILVLAADLIND1APSLSSALIAHP--QP
          	.:: ** .*:.*  .  **** : : ..: **:*** .**::  .*:*:.*::     :*

MG04827.1 	YRQLLPLLAQSSGPEDPIPLLTSHVLVNLIAG---SRDESDLTLQKALPVILSYLSTLTK
NCU02746.1	YKNILPLLAHSTNTEDPIPLLTSIVLVRLMAG---SRDESTATAEKALPMLFSYLGSLTK
FG01152.1 	YRHFLPLLN-STNAEDPIPLLTAHALTTLMAL---ARDESRSTLQ-ALPVIFTYLSGLAK
AN1211.1  	YKPFLPLLRHSTNAEDPIPLLTSNFLTNLVSTSITSSSKSTPQDEEALPQLYHYLSTLTQ
          	*: :****  *:..********:  *. *:: : :: .:*    :.*** :  **. *::

MG04827.1 	SSDAGLQDIGVQEYSTVLFGSKSRSQFWAQRSETVAPLINILRAAAGSS-NGDANG-IRS
NCU02746.1	NSDAGLQDIGVQEYSQLLYSRVCRQQFWKQRSETVGPLIDILRAAAGIS-GGDASANLWS
FG01152.1 	SNDAGLQDIAVQEYSGLLFGHAAREQFWNQQSETIAPLIKILQTAANIGNGGSSSASLWS
AN1211.1  	NQDSGLQDIGVQGLSTLLRTSRARQLFWAQRKETVDPLVEILRAAAGSKDSGS------S
          	..*:*****.**  * :*    .*. ** *:.**: **:.**::**.  ..*.      *

MG04827.1 	GSMRGGP--VEGSIGGGVGLQLLYHVLLVMWQLSFEAEDVGCDLDD2YVSDSFLREYNII
NCU02746.1	GTTAPPRSGFEASVGGGVGLQLLYHVLLVLWQLSFEAADIGDDLNN2--------EYDIV
FG01152.1 	GNTGTASTGFGGSLGGGVGLQLLYHALLVIWQISFEAEEIGDDLND2--------EYDIV
AN1211.1  	TTLAGSSRGVDVGIAGGVGLQLLYRVLLVLWQLSFEGELIGDELQE2--------DHEIL
          	 .      ..  .:.*********:.***:**:***.  :* :*::         :::*:

MG04827.1 	QLYTQLLKLSPKEKTTRLIVSTLYNLLSANRSSLLPVASFARLPSLLSNITSRQLTDPDL
NCU02746.1	LLYTHLLRLSPKEKTTRLVIATLYNLLSNNQNSLLPTAVLARLPQLLQNLSSRQFTDPDL
FG01152.1 	LLYTHLLRLSPKEKTTRLILSTLYNLLDKNQKSLLPTAVLARLPALLENISGRHLTDPDL
AN1211.1  	QLYSHLLRLSPKEKTTRLLLATLRNLLSFNRTTLLPVAVFVRLPALLSTISGRHLTDPDL
          	 **::**:**********:::** ***. *:.:***.* :.*** **..::.*::*****

MG04827.1 	QEDLQSLKDMMDEYTATKTTFDEYVAEVTNGHLRWSPPHRSQTFWAENARRILDENNAGV
NCU02746.1	VEDRDKLRELVEEYTKTKTTFDEYVAEVNSGHLRWSPPHRSSVFWAENARKILDYENGEI
FG01152.1 	LEDLSKLKEMLEEYTKTKTTFDEYIAEVQSGHLRWSPPHRNTVFWAENARKILEFENGTI
AN1211.1  	LEDLKYLSEMLDEYTKTQTTFDQYAAELQSGHLRWSPPHRNPTFWKENARRILDENSGAL
          	 ** . * ::::*** *:****:* **: .**********. .** ****:**: :.. :

MG04827.1 	VRQLAEIMKKPWDNDKQVLAIACNDIGALVREVPEKRGQLERLGLKTRIMELMGEADENV
NCU02746.1	VRKLAEIMQKPWENDKAVLAIACNDVGCLVREVPEKRSQLERYGLKRRIMELMGEADENV
FG01152.1 	PRKLAEIMQQPWDNDKQVLAIACNDVGCLVKEVPEKRYQLEKVGLKRRIMELMQSDDENV
AN1211.1  	PKKLKEIISKSWENDKQVLAIACNDVGHLVKELPERRGQLEKLGFKTRVMELMADKDESV
          	 ::* **:.:.*:*** ********:* **:*:**:* ***: *:* *:**** . **.*

MG04827.1 	RWESLKALGGWLKYSFENNK
NCU02746.1	RWESLRALGGWLQYSFDTK-
FG01152.1 	RWESLRALGGWLKYSFEQK-
AN1211.1  	RWESLRAVGEWLRYTFDD--
          	*****:*:* **:*:*:
```
